# Public Knowledge About Emergency Care—Results of a Population Survey From Germany

**DOI:** 10.3389/fpubh.2021.787921

**Published:** 2022-01-07

**Authors:** Olaf von dem Knesebeck, Sarah Koens, Ingmar Schäfer, Annette Strauß, Jens Klein

**Affiliations:** ^1^Institute of Medical Sociology, University Medical Center Hamburg Eppendorf, Hamburg, Germany; ^2^Department of General Practice and Primary Care, University Medical Center Hamburg Eppendorf, Hamburg, Germany

**Keywords:** knowledge, emergency care, health literacy, social inequalities, population, Germany

## Abstract

**Background:** Knowledge and beliefs about health and health care are part of the general concept of health literacy. Studies demonstrated that large parts of the population report inadequate health literacy. There are only few studies specifically addressing public knowledge and beliefs about emergency care. We examine magnitude and social variations of public knowledge about emergency care in Germany.

**Methods:** Analyses make use of a telephone survey conducted in Hamburg, Germany. Random sample consisted of 1,207 adult respondents. We asked whether the respondents know various emergency care services. Moreover, capabilities of dealing with an emergency case were assessed. Sex, age, education, and migration background were introduced as predictors into regression models.

**Results:** 98% of the respondents stated to know the rescue service, while 74% knew the medical on call service and 49% were aware of an emergency practice nearby. About 71% of the interviewees said it was easy for them to find out whom to turn to in a case of a medical emergency. Fewer respondents found it easy to evaluate when to use emergency medical services and to evaluate whether a health problem is a medical emergency. Knowledge and capabilities were positively associated with education and negatively related to migration background.

**Conclusions:** This study indicates a lack of public knowledge about emergency care and social inequalities in public knowledge according to education and migration status. Findings suggest that interventions are needed to improve public knowledge and that considering social inequalities should be a basic principle for such interventions.

## Introduction

Knowledge and beliefs about health, illness and health care are part of the general concept of health literacy. Health literacy indicates the degree to which individuals have the capacity to obtain, process, and understand health information and services needed to make appropriate health decisions (1). Population based studies from European countries, including Germany, demonstrated that between one and two thirds of the population report inadequate or problematic health literacy (2). Limited health literacy was found to be associated with increasing age, low education, and migration background (3), indicating social inequalities in health literacy.

There is a growing body of literature from the U.S. and Europe showing that low levels of public health literacy are associated with more frequent utilization of curative health services (4, 5). In this regard, low health literacy may contribute to the overcrowding of emergency departments with patients suffering from low acuity conditions observed in some countries (6, 7). Accordingly, some studies demonstrated an association of low health literacy and frequent utilization of emergency medicine (8, 9). However, not all analyses confirmed this association (10, 11).

There are only few studies specifically addressing public knowledge and beliefs about emergency care (12). In this regard, results of a German study showed that public knowledge regarding different options for treatment of acute medical events and competence to assess urgency seems to be insufficient (13). However, this study was conducted before the COVID-19 outbreak that changed awareness and utilization of emergency care (14–16). Moreover, it did not analyse social inequalities in public knowledge about emergency care.

Based on a population sample from a large city in Germany (Hamburg), we will explore the following research questions: Which emergency care services does the public know? How does the public estimate the capabilities of dealing with an emergency case? Are there social variations (according to sex, age, education, and migration status) in public knowledge and reported capabilities?

## Methods

### Study Design and Sample

Analyses make use of a cross-sectional telephone survey conducted in Hamburg, Germany in winter 2020/2021 *via* CATI (computer assisted telephone interviews). The adult sample (people aged 18 years and older) was randomly drawn using all possible telephone numbers in Hamburg, including non-registered numbers via random digital dialing. Repeated calls were made by trained interviewers of a professional survey research institute on different weekdays. To randomly select the target person in the households, the Kish selection grid was used (17). Sample size calculation was based on a vignette design applied in the study. These vignettes were not used in the present analyses. Sample consisted of 1,207 respondents. As there are different approaches for the definition of eligibility in telephone surveys, different response rates can be calculated (18, 19). Accordingly, response rate (RR) in this survey varies between 11.8 and 43.8% [American Association of Public Opinion Research RR_3_ (18) 16.4%]. To improve the quality of the sample, it was weighted for sex, age and educational level. Comparisons with official statistics from Hamburg (20) indicated that the weighted sample did not significantly differ from the general adult population regarding the distribution of sex, age, and level of education. The study was approved by the Local Psychological Ethics Committee at the Center for Psychosocial Medicine, University Medical Center Hamburg (No. LPEK-0200). Respondents gave their informed consent for the participation and the use of their data. Consents and refusals were documented by the interviewers.

### Measures

In the German health care system, patients can either contact the medical on call service (also known as “116117” referring to the telephone number) or utilize emergency medicine [accident and emergency departments, emergency practices, rescue service (telephone number 112)] in urgent cases. To assess knowledge about emergency care services, we asked whether the respondents know: 1. the rescue service, 2. the medical on call service, and 3. an emergency practice nearby (yes/no). We did not include emergency departments as these are often overcrowded (21) and we were particularly interested in knowledge about alternative emergency care services. Capabilities of dealing with an emergency case were measured by three newly developed questions that were inspired by the European Health Literacy Survey Questionnaire (HLS EU Q, 22). Respondents were asked how easy/difficult it is in their opinion 1. to find out whom to turn to in a case of a medical emergency, 2. to evaluate when to use emergency medical services, and 3. to evaluate whether a health problem is a medical emergency. Response categories were “very difficult,” “rather difficult,” “rather easy,” and “very easy.” A principal component analysis revealed that all three items loaded on one factor (Eigenvalue 1.84; explained variance 61.37%; loadings 0.72 to 0.82; Cronbach's Alpha 0.68). For the multivariate analyses, a sum scale was calculated with higher scores indicating higher reported capability of dealing with emergency cases.

Sex, age, education (in years of schooling), and migration background (no, 1st generation, 2nd generation) were introduced as social factors. A person has a migration background, if the person him-/herself or at least one parent was born abroad. Respondents with a migration background who were born in Germany are considered as 2nd generation migrants, while those with an own migration experience are subsequently termed 1st generation migrants.

### Analyses

Descriptive analyses were conducted to show the proportion of respondents knowing the emergency care services and reporting capabilities of dealing with emergency cases. Chi-square tests were conducted to analyse bivariate associations of knowledge and capabilities (single items) with the social factors. Moreover, logistic regression models were calculated to analyse social variations in knowledge. Odds ratios, 95% confidence intervals (95% CI), significances (p), and Nagelkerkes *R*^2^ were documented. In terms of the sum scale measuring capability of dealing with emergency cases, a linear regression analysis was conducted to explore associations with the social factors. Standardized regression coefficient (beta), *p*-values, and explained variance (*R*^2^) were shown. Results with *p* < 0.05 were considered statistically significant. All analyses were performed using IBM SPSS Statistics 26 (23).

## Results

In terms of the social characteristics of the sample, 51.5% was female, mean age was 48.6 years (SD 18.76), and 49.4% had 12 years of schooling or more. About three quarter (77.3%) had no migrant background, while 11.7% belonged to the 1st generation of migrants and 10.9% to the 2nd generation.

A large majority of 98% of the 1,207 respondents stated to know the rescue service (112), while 74% knew the medical on call service and 49% were aware of an emergency practice nearby. About 71% of the interviewees said it was very or rather easy for them to find out whom to turn to in a case of a medical emergency. Fewer respondents (56.2%) found it easy to evaluate when to use emergency medical services and to evaluate whether a health problem is a medical emergency (43.2%).

Table 1 shows the bivariate associations of knowledge and capabilities (single items) with the social factors. There was only one significant association with sex: Knowledge of the medical on call service was more pronounced among female respondents. All three items indicating capabilities of dealing with an emergency case were negatively associated with age. Education was significantly associated with knowledge and capabilities, with the exception of knowledge of an emergency practice nearby. In terms of migration background, significant associations with knowledge of the rescue service and the medical on call service emerged.

**Table 1:** Knowledge of emergency care services and capability of dealing with emergency cases according to social factors: bivariate analyses (*N* = 1,207).

		**Do you know… (% yes)**			**How easy/difficult is it in your opinion… (% easy/rather easy)**		
		**The rescue service (tel. 112)?**	**The medical on call service (tel. 116117)?**	**An emergency practice nearby?**	**To find out whom to turn to in a case of a medical emergency?**	**To evaluate when to use emergency medical services?**	**To evaluate whether a health problem is a medical emergency?**
Sex	Male (*n =* 585)	98.1	67.7	47.4	73.0	56.7	41.7
	Female (*n =* 622)	97.9	80.0	50.2	68.9	55.6	44.7
	*p* ^*^	0.794	**<0.001**	0.329	0.119	0.711	0.301
Age (years)	18–40 (*n =* 455)	97.4	70.4	48.1	76.1	62.6	46.6
	41–60 (*n =* 419)	98.6	76.4	53.3	70.7	55.4	45.3
	> 60 (*n =* 332)	98.2	76.2	44.6	63.7	47.6	35.6
	*p*	0.430	0.076	0.052	**0.001**	**<0.001**	**0.006**
Education (years)	≤ 9 (*n =* 316)	98.1	64.6	47.3	58.0	47.0	31.4
	10 (*n =* 275)	99.6	78.9	53.8	69.0	60.8	48.3
	≥ 12 (*n =* 574)	97.0	76.7	46.2	80.1	58.9	47.8
	*p*	**0.044**	**<0.001**	0.108	**<0.001**	**0.001**	**<0.001**
Migration background	No (*n =* 915)	98.7	80.0	48.4	71.4	54.8	43.5
	2nd gen. (*n =* 129)	94.6	55.0	51.2	70.9	56.0	35.9
	1st gen. (*n =* 141)	97.2	54.6	47.5	68.3	61.9	44.1
	*p*	**0.008**	**<0.001**	0.811	0.757	0.299	0.252

* *Pearson's Chi-square test; significant differences (p < 0.05) are bold*.

Logistic regression analyses revealed that, compared to respondents without a migration background, those who belonged to the 2nd generation were significantly less likely to know the rescue service (Table 2). In terms of the medical on call service, knowledge was significantly more pronounced among women, and people with higher education, while there was less knowledge among respondents with a migration background. Knowledge of an emergency practice nearby showed no significant associations with the social factors under study. The goodness-of-fit based on the Hosmer-Lemeshow-Test indicated good model fits [knowledge of rescue service (*p* = 0.187), medical on-call service (*p* = 0.221), emergency practice (*p* = 0.586)]. Multivariate analyses of the association between social factors and the sum scale (Table 3) showed, that capability of dealing with emergency cases significantly increased with education.

**Table 2:** Knowledge of emergency care services according to social factors: logistic regression models.

	**Rescue service (tel. 112) (*****N*** **=** **1,178)**		**Medical on call service (tel. 116117) (*****N*** **=** **1,178)**		**Emergency practice nearby (*****N*** **=** **1,177)**	
	**OR (95% CI)**	***p***	**OR (95% CI)**	***p***	**OR (95% CI)**	***p***
Age^*^						
41–60	1.602 (0.550–4.665)	0.387	1.189 (0.857–1.650)	0.300	1.150 (0.873–1.514)	0.320
> 60	0.905 (0.304–2.691)	0.858	1.432 (0.990–2.072)	0.056	0.817 (0.600–1.112)	0.199
Sex (female)	0.976 (0.422–2.255)	0.955	**2.058 (1.554–2.727)**	**<0.001**	1.154 (0.914–1.457)	0.228
Education^**^						
10	4.040 (0.563–28.988)	0.165	**2.135 (1.437–3.173)**	**<0.001**	1.208 (0.867–1.682)	0.265
≥ 12	0.658 (0.236–1.833)	0.424	**2.172 (1.550–3.045)**	**<0.001**	0.854 (0.636–1.146)	0.293
Migration background^***^						
1st generation	0.491 (0.153–1.577)	0.232	**0.292 (0.198–0.430)**	**<0.001**	0.931 (0.649–1.336)	0.699
2nd generation	**0.287 (0.106–0.775)**	**0.014**	**0.313 (0.210–0.467)**	**<0.001**	1.145 (0.786–1.666)	0.481
Nagelkerkes *R*^2^	0.066		0.136		0.013	

*OR, odds ratio; CI, confidence interval; p, significance;*

* *reference 18–40 years*,

** *reference ≤ 9 years*,

*** *reference no migration background, significant associations (p < 0.05) are bold*.

**Table 3:** Social factors and capability of dealing with emergency cases (sum scale): linear regression model (*N* = 1,103).

	**Standardized coefficient (Beta)**	***p***
Age^*^		
41–60 years	−0.023	0.494
> 60 years	−0.067	0.052
Sex (female)	0.033	0.262
Education^**^		
10 years	**0.153**	**<0.001**
≥ 12 years	**0.219**	**<0.001**
Migration background^***^		
1st generation	0.003	0.915
2nd generation	−0.042	0.163
*R* ^2^	0.047

*p = significance*,

* *reference 18–40 years*,

** *reference ≤ 9 years*,

*** *reference no migration background, significant associations (p < 0.05) are bold*.

## Discussion

Based on a population sample from a large city in Germany (Hamburg), we found that almost all of the respondents (98%) stated to know the rescue service (“112”), while there was a lack of public knowledge regarding the medical on call service (“116117”) and emergency practices nearby. About 25% of the respondents did not know the former and about 50% did not know the latter. We also found a lack of capabilities of dealing with emergency cases. This held especially true for the decision when to use emergency medicine and the identification of a health problem as an emergency case. Overall, knowledge and capabilities tend to be more pronounced among women, while associations with age were inconsistent. Finally, we predominantly found positive associations with education and negative associations with migration background.

In a previous German study (13), knowledge of the rescue service and the medical on call service was very similar, whereas the authors did not ask about emergency practices. A lack of knowledge and capabilities with regard to emergency care can be considered as an indicator of limited health literacy. In this regard, the present analyses aimed to make a contribution to research on public emergency literacy. Some studies indicate that a low health literacy is associated with more frequent as well as inadequate health care use, including emergency care (5, 9, 24). In Germany, emergency care is provided by statutory health insurance physicians as well as by ambulance services and hospital emergency departments (21). If symptoms are life threatening, patients can either call the rescue service or go to an emergency department. If symptoms are urgent but not life threatening, patients can either contact the medical on call service or go to an emergency practice. The medical on call service can be used to ask for advice and to make medical appointments, and alternatively, a home visit by the doctor can be arranged. As many respondents in our study did not know the medical on call service or an emergency practice, it seems likely that they attend emergency departments or contact the rescue service although their symptoms may not be life threatening. This is supported by a study of Scherer et al. (21) showing that more than half of patients attending emergency departments assessed their treatment urgency as low and therefore did not meet the definition of an emergency.

Knowledge of the medical on call service as well as reported capability of dealing with emergency cases were more pronounced among better educated people, indicating educational inequalities in public emergency literacy. Previous studies on the broader concept of health literacy found respective inequalities in Germany (3) and in other European countries (2). Limited health literacy was also demonstrated for people with migration background (3) and there is evidence for higher use of emergency services among migrants compared to non-migrants across Europe (25). A recent German study found that migrants show lower odds of adequate emergency department use compared to non-migrants (26). Our results indicate that a lack of knowledge especially of the medical on call service among people with a migration background may in part explain these differences. In the present analyses, 1st and 2nd generation migrants were differentiated. In this regard, respondents with a migration background who were born in Germany are 2nd generation migrants, while those with an own migration experience are 1st generation migrants. Our results showed only weak differences between these two generations. One could have expected that knowledge about emergency care services is even more limited among 1st generation migrants. However, our results are in line with the study mentioned above (26) which also found small differences between 1st and 2nd generation migrants in terms of adequate emergency department use. Moreover, a recent study that compared health literacy in Germany before and during the COVID-19 pandemic found out that improvements in health literacy were particularly strong among 1st generation migrants (27).

Although knowledge and capabilities tend to be more pronounced among women, associations do not reach statistical significance in most cases. There is one exception: Significantly more women report to know the medical on call service. This service was introduced in Germany for treatment outside normal appointment times and it became more known during the COVID pandemic as this is one officially recommended option to get help in case of a potential COVID-19 infection. Obviously information about this service reached more women than men.

The present study has some limitations that need to be considered. Analyses are based on a random population sample from a large city in Germany (Hamburg). Thus, findings refer to an urban population that may differ from other regions in Germany and elsewhere. Although the response rate is adequate for a telephone survey (28) and the sample is similar to official statistics regarding the distribution of socio-demographic characteristics, we cannot rule out a selection bias due to non-response. As there was no validated measure for emergency literacy available, we developed three items to assess reported capabilities of dealing with an emergency case. While we took measures of general health literacy into account (22) and psychometric properties of the sum scale (Cronbach's Alpha 0.68) seem adequate, indicators of emergency literacy need to be further developed and tested. Finally, in terms of the analyses of social variations in knowledge of the rescue service, one has to keep in mind that only 2% (*n* = 24) did not know the service and hence, empty cells occurred in the classification tables on the main diagonal. Therefore, the estimates should be interpreted with caution.

Despite these limitations, this study indicates a lack of public knowledge about emergency care and social inequalities in public knowledge according to education and migration status. These findings suggest that interventions are needed to improve public knowledge and capabilities. In this regard, action plans to promote health literacy have been developed in some countries (1, 29). Our results underline that considering social inequalities should be a basic principle in implementing such action plans.

## Data Availability Statement

The raw data supporting the conclusions of this article will be made available by the authors, without undue reservation. Data are available on reasonable request.

## Ethics Statement

The study was approved by the Local Psychological Ethics Committee at the Center for Psychosocial Medicine, University Medical Center Hamburg (No. LPEK-0200). Respondents gave their verbal informed consent for the participation and the use of their data. Consents and refusals were documented by the interviewers.

## Author Contributions

OK, JK, and IS designed the study. SK and JK conducted the analyses. OK interpreted the data and drafted the manuscript. JK, AS, SK, and IS critically revised the manuscript and approved the final version. All authors contributed to the article and approved the submitted version.

## Funding

This study was funded by the Federal Ministry of Education and Research, Germany (Grant No. 01GY1912).

## Conflict of Interest

The authors declare that the research was conducted in the absence of any commercial or financial relationships that could be construed as a potential conflict of interest.

## Publisher's Note

All claims expressed in this article are solely those of the authors and do not necessarily represent those of their affiliated organizations, or those of the publisher, the editors and the reviewers. Any product that may be evaluated in this article, or claim that may be made by its manufacturer, is not guaranteed or endorsed by the publisher.
